# Molecular characterization and tissue tropism of an Iraqi field isolate of fowl adenovirus serotype 8a in broiler chickens

**DOI:** 10.1371/journal.pone.0348443

**Published:** 2026-05-11

**Authors:** Mohammed Abdullah Hamad, Ahmed J. Alfahdawi, Basim Mohamed Manswr

**Affiliations:** 1 Department of Biotechnology, College of Applied Sciences, University of Fallujah, Iraq; 2 Department of Pathological Analysis, College of Applied Sciences, University of Fallujah, Iraq; 3 Department of Pathology, College of Veterinary Medicine, University of Diyala, Iraq‌‌; PRISM CRO, PAKISTAN

## Abstract

**Background:**

Inclusion body hepatitis is still a threat to broiler farms and recent findings indicate that FAdV-8a is re-emerging in several areas. Limited data are available on circulating strains in the Middle East. The objectives of this work were to characterize an Iraqi field isolate and to study its effect on various tissues in experimentally infected broilers.

**Methods:**

The experiment was conducted on a total of 95 Cobb-500 chicks that were randomly assigned into infected and control groups. At 14 days of age, the chicks were inoculated with the isolate ocularly. Birds were necropsied 21 days post-infection, with organ weights, gross and histopathological findings, and qPCR data targeting the hexon gene used to assess the infection.

**Results:**

Infected birds had increased liver and spleen sizes indicated by an increase in the organ/body weight ratio. The liver was pale and swollen, spleens were increased in size and dark, the superficial area of the kidney presented a diffuse pallor while the changes in the heart were negligible. Microscopically, there were severe hepatic degeneration and necrosis, noticeable splenic lymphoid depletion, and tubular injury in the kidneys consistent with secondary changes, with mild cardiac alterations. These results were further confirmed by qPCR analysis, which demonstrated relatively high viral genome copies in liver and moderate copies in spleen, whereas minimal copy numbers were found in heart.

**Conclusion:**

The hepatic involvement was the predominant disease picture induced in chickens by the Iraqi FAdV-8a isolate, with associated splenic changes and mild renal histological alterations and slight cardiac involvement. The infection was non-fatal to the controlled animals, indicating low virulence. These results provide important regional information that can help direct monitoring and serotype-specific prevention strategies.

## Introduction

Fowl adenoviruses (FAdVs) are non-enveloped viruses with double-stranded DNA genomes and they are classified within the genus *Aviadenovirus*. These viruses are widely distributed in commercial poultry populations and they have been associated with different clinical outcomes. In many cases infection remains subclinical, however in other situations the virus may lead to clear disease and economic losses. Field observations during recent years indicate that FAdV infections are still being reported in different parts of the world, and in some regions the predominant serotypes appear to change with time, together with differences in pathogenic characteristics that may depend on geographical location and production conditions [1–3]. Among the diseases associated with FAdV infection, inclusion body hepatitis (IBH) is considered one of the important conditions affecting broiler production. The disease is usually characterised by a sudden increase in mortality, often reported in the range of 10–30%. This may also be accompanied by poor weight gain, together with characteristic hepatic lesions that can be observed during necropsy and later confirmed by histopathological examination [4–6]. IBH is most frequently associated with viruses belonging to FAdV species D and E including serotypes 2, 8a, 8b and 11. These serotypes mainly affect the liver, although lesions may also be observed in spleen and kidneys, and less consistently in the heart when infection becomes more systemic, but this is not always observed in all cases [7–9]. Evidence from regional studies indicates that the distribution of these serotypes is not the same in all locations and may vary between countries. For example, investigations conducted in Bangladesh reported that serotype 11 (species D) and serotype 8b (species E) were the predominant causes of IBH outbreaks in both broiler and layer flocks. The mortality levels in these outbreaks also appeared to be affected by production conditions as well as the route of exposure [4]. More broadly, surveillance programmes indicate that the relative prevalence of FAdV serotypes is not fixed and may change over time across geographic regions and poultry production systems, therefore continued epidemiological monitoring together with local characterisation of circulating strains becomes important for better understanding of disease behaviour [10]. FAdVs may be transmitted by both horizontal and vertical routes. The horizontal spread usually occurs through the faecal–oral pathway. Vertical transmission can also occur, when infected breeder hens pass the virus to their progeny. Because of these two possibilities, the virus may continue to circulate in breeder as well as broiler flocks. This is more likely when biosecurity measures are not strict or when vaccination programmes are not consistently applied. Under such conditions, the infection can persist within poultry populations for long periods [11,12]. In addition, several studies indicated that vertical transmission may contribute substantially to outbreaks in young broilers, with clinical disease often reported between the third and fifth week of life, which corresponds to decline of maternal antibodies in many flocks [13]. The pathogenesis of FAdV infection has been extensively studied through experiments, which consistently reveal that the major target organ is the liver. Hepatocellular infection is often accompanied by focal necrosis and intranuclear inclusion bodies. These lesions of the liver are usually correlated with different levels of lymphoid depletion. Quantitative studies show that the viral load is highest in the liver, with moderate levels found in the spleen and lower levels in the other organs examined; yet this may depend on the strain of virus [8,14,15]. As the molecular diagnostics methods become more sophisticated, the diagnosis and typing of FAdV strains become more accurate. Sequencing of loop-L1 of the hexon gene serves this purpose very well. Studies from countries including Egypt and Iraq applied this approach and reported notable genetic diversity among circulating FAdV strains, together with the appearance of new field isolates within the region [16–18]. Comparable molecular strategies have also been employed to characterise other poultry viruses and to better understand their epidemiology in commercial flocks [19]. Despite these developments, experimental studies specifically addressing FAdV-8a under controlled conditions remain comparatively limited, particularly in the Middle East. Only a limited number of investigations integrated pathological assessment with histopathology and quantitative molecular evaluation of viral distribution across infected organs [20,21]. In the present study, broiler chickens were experimentally infected with a field isolate of FAdV-8a recovered from Iraqi broiler flocks, and the aim of this work was to assess pathological changes, histopathological lesions and distribution of viral DNA in major organs following infection, since evaluation of these parameters under controlled conditions may provide additional insight into tissue tropism and pathological behaviour of FAdV-8a and also contribute region-specific data that may support future monitoring and control efforts.

## Materials and methods

### Ethical approval

All procedures carried out in this study were reviewed and approved by the Institutional Animal Care and Use Committee (IACUC) at Uruk Private Laboratories in Baghdad, Iraq (Approval No. UPL-IACUC-2022–014; issued 15 December 2022). The work was performed under the direct oversight of the laboratory manager, Dr. Ameer Hadi Farhan, PhD. Animal handling, housing, inoculation, and sampling followed internationally accepted guidelines for the care and use of animals in research and complied with relevant Iraqi regulations. As the study did not involve human participants or any human-derived material, no additional ethics review or informed consent requirements were necessary.

### Study design and experimental overview

The experiment was designed as a controlled challenge to help understand how a field isolate of fowl adenovirus serotype 8a (FAdV-8a) behaves in broiler chickens and how it spreads within different organs. The work was shared between two laboratories: Uruk Private Laboratory in Baghdad, where the birds were infected and the molecular work was carried out, and Wahj Al DNA Laboratory, also in Baghdad, which handled all of the histology. In total, 95 Cobb-500 chickens were obtained from different hatcheries on the first day of their life. All birds that were purchased went through an initial inspection to ensure that they are healthy enough for use in the experiment. Next, all subjects were randomly allocated into two groups: one with 50 infected animals and another with 45 uninfected animals. The total number of birds at the beginning of the experiment was fixed to establish a stable model of infection at the flock level. This design also allowed continuous monitoring of clinical signs, survival, and overall infection dynamics at the flock level throughout the experimental period, in accordance with the approved ethical guidelines. A representative subset of birds from each group (n = 7 per group) was selected for terminal sampling at 21 days post-infection for detailed pathological, histological, and molecular analyses. The remaining birds were maintained for monitoring of clinical signs, survival, and overall infection outcome at the flock level throughout the experimental period and were humanely euthanized at the completion of the study according to the approved ethical protocol. No mortality or severe clinical signs were observed among the remaining birds during the experimental period (Supplementary S1 Table). The two groups were reared under the same conditions in controlled temperature, ventilation and light (23 hour light; 1 hour dark) with ad libitum access to feed and clean water in order to eliminate any management discrepancy that could influence the results. The inoculation was performed at 14 days of age. Sampling was carried out one time only at 21 days post-infection (at a chicken age of 35 days). It took 35 days to conduct the experiment (from day old to 35 days old, 21 days post infection). Those birds were then investigated for gross lesions, organ to body weight ratio, histopathological changes and viral DNA copy number. This one point in time strategy was chosen to monitor the infection at a time when it was fully developed while maintaining identical conditions for all birds. Humane endpoints were predefined, and all birds were euthanized at the scheduled study endpoint. Each of the birds of the study was observed closely for the presence of extreme and anti-morbidity distress for two times a day. According to the study, euthanasia was mandatory if the birds were remarkably apathetic and unresponsive to any of the stimuli, are trapped in a combination of starvation and dehydration, severe pain from respiratory distress and/or are profoundly hypotonic to the extent of being unable to stand, and are anticipated to lose more than twenty percent of their original weight. Euthanasia was planned and carried out for all of the birds meeting any of these. The birds remained alive throughout the study, and none required euthanasia outside the pre-established schedule. All birds were humanely euthanized at the predefined sampling point (21 days post-infection). The endpoints and study design were pre-registered with Uruk Private Laboratories (Approval no UPL-IACUC-2022–014) Institutional Animal Care and Use Committee (IACUC), which led to the review and approval of the study. All procedures were carried out by trained personnel under the supervision of Dr. Ameer Hadi Farhan.

### Birds, housing conditions, and eligibility criteria

Day‐old 95 Cobb‐500 broiler chicks (from commercial flocks) were transported from hatch day to research station. On arrival of the chicks, a cursory physical examination was conducted in order to ensure that each bird appeared healthy and was eligible for the study. Only bright, active chicks with body weight close to 10% above the average of the batch were selected. Any chick that appeared weak, with a visible deformation or presenting signs of injury was discarded. Birds of both the experimental groups were kept under environmental conditions in controlled rooms using standardized husbandry methods, which provided unrestricted access to food and water. Each room had its own heating, ventilating, and lighting system; hence, similar conditions prevailed for both the groups. During the first week, a temperature of 32–34°C was kept and gradually decreased until about 24°C according to a standard broiler management practice. The photoperiod was maintained at 23 hr light:1 hr dark during the experiment. The feed and drinking water were available ad libitum, while routine husbandry procedures were performed similarly for the infected as well as the control group so that no unwarranted differences are introduced. There were 95 birds in total. Fourteen birds (seven infected and seven control) were humanely euthanized at the predefined experimental sampling point (21 dpi) for tissue collection and analysis. No birds required unplanned humane euthanasia prior to this scheduled endpoint. The remaining 81 birds were euthanized at the completion of the study according to the approved protocol. No birds were found dead and no severe clinical signs were observed during the experiment.

### Virus isolation, characterization, and inoculum preparation

Suspicious field samples regarding fowl adenovirus were taken in broiler chicken farms presenting clinical signs that typically bring suspicion of inclusion body hepatitis. These samples were handled in aseptic ways and virus was isolated on primary chicken embryo liver (CEL) cell cultures. We observed for the cytopathic effects associated with adenovirus once cultures were established, and these changes indicated that virus had established an infection in cells. Once growth was confirmed, the isolate was sub-cultured to generate a working stock of sufficient size for use in subsequent experiments. In order to confirm that the isolate was FAdV-8a, the sequence of the hexon gene was obtained as this is often used for determining adenovirus serotypes. Upon sequencing verification of the serotype, the viral suspension was titrated by TCID₅₀. The concentration of the final inoculum was brought to 10⁴.⁵ TCID₅₀/mL. This inoculum was used to infect all the birds in the challenge group, in order to ensure that every bird received equivalent doses.

### Experimental infection and sampling schedule

The 95 chicks were randomly assigned into two groups, with 50 (infected group) subjected to infection and 45 (control group). At 14 days of age, birds in the infected group were inoculated with 0.2 mL of the prepared FAdV-8a suspension (10⁴.⁵ TCID₅₀/mL) via the ocular route (0.1 mL per eye). Control birds were processed similarly but received sterile phosphate-buffered saline instead of virus. Post-inoculation, the birds were monitored daily for general behavior, feed intake and overt signs of disease. Sampling occurred once, at day 21 post-infection (35 days of age). After light anesthesia (lidocaine HCl 2% (20 mg/ml) injectable solution), on the day of sampling, seven birds from each group were slaughtered by cervical dislocation. Immediately following euthanasia, body weight of each bird was recorded and before removing the liver, spleen and heart for organ-to-body weight calculations, qPCR, and histopathology. Kidneys were also obtained, but only for histopathological analysis as their involvement was identified at necropsy. This 21-day post-infection sampling time point was selected to allow us to study the infection at a uniform stage in all of the birds.

### Organometric measurements

At 21 dpi, all birds were weighed immediately before the beginning of necropsy. Once the body cavity was opened, the liver, spleen and heart were extracted and cleaned, removing any adhered tissues. Excess moisture was gently dabbed from the organs to allow the weights to be measured accurately and reproducibly. Thereafter, organs were weighed individually on a calibrated electronic balance. To assess whether enlargement was triggered by the infection, organ weight was expressed relative to body weight for each bird. The same measurement devices were used for all birds, and the measurements from infected birds were compared with those from non-infected birds to ensure fair and consistent comparisons between both groups.

### DNA extraction and quantitative PCR (qPCR)

To detect FAdV-8a, liver, spleen, and heart tissues were collected from five randomly selected birds from each group at 21 days post-infection (dpi) for quantitative PCR analysis. ~ 25 mg of tissue from different organs were homogenized and treated with the Patho Gene-spin DNA/RNA Extraction Kit (iNtRON Biotechnology, Korea; Cat. No. 17054). The extraction was done as per the manufacturer’ s manual. Following DNA isolation, concentration and purity were assessed with a NanoDrop 2000 spectrophotometer (Thermo Fisher Scientific, USA). Subsequently, the concentration of all samples was normalized to facilitate a comparison of qPCR results. The qPCR primers were designed from the conserved region of the hexon gene (the Hexon-F 5′-TGG CTA CAT GTC CCG GAA C-3′, Hexon-R 5′-CGC ATG TTG GGT TCA GGT C-3′) as described previously for detection of FAdVs. Each reaction was performed in a 20 µL volume with 2 × iQ SYBR® Green Supermix (Bio-Rad, USA; Cat. No. 1708880), as well as 0.5 µM of each primer, 2 µL DNA template, and nuclease-free water. The amplification was performed in a CFX96 Touch Real-Time PCR Detection System (Bio-Rad Laboratories, Inc., USA). Cycling was composed of a 3-min initial denaturation at 95°C and then 40 cycles of the following sequence: (i) 95°C for 15 s and (ii) annealing/extension at 60°C for 30 s. A melt curve was included to verify that the amplified product was the single product. Samples with Ct values equal to or greater than 40 were considered negative for viral DNA. Viral copy numbers were calculated using the standard curve that was generated from ten-fold dilutions of purified viral DNA.

### Gross pathology examination

All birds included in the sample collection were subjected to a complete gross post-mortem examination 21 days post infection. After euthanasia, the body cavity was opened gently and no damage to liver, spleen, heart and kidney was caused by opening of the body. In the sections, size, colour and consistence of every organ were reported as well as any evident lesion. Since adenoviral infections tend to be associated with specific slips and stains, extra care was taken for signs such as hepatomegaly or pallor (mottling), splenomegaly and renal swelling/darkening. The surface dullness, congestion and mild edema were checked in the heart as well. When characteristic lesions were visually present, organs that were visibly affected were photo-documented using a high-resolution digital camera in identical lighting conditions for all samples. Everything was documented in advanced and compared afterwards with histology and qPCR results in relation to what could be seen (or not) so it would be possible to make a connection between the macroscopical findings and the microscopy and molecular findings.

### Histopathological examination

Immediately following necropsy, small sections of the liver, spleen, heart and kidney were taken from each bird sampled at 21dpi. Tissues were immersed in 10% neutral buffered formalin as soon as possible and allowed to fix for a minimum of 48 hours. For fixation, the samples were subjected to dehydration, clearing and paraffin embedding, after which followed standard processing. Sections were then sliced at approximately 4–5 µm on a rotary microtome. The sections were mounted onto microscope slides and stained with hematoxylin and eosin using standard methodology. The slides viewed under a light microscope (Olympus CX43, Japan) were inspected for the type of alterations which should normally show up in adenovirus infection. These were foci of either hepatocellular degeneration or necrosis, inflammatory cell infiltrate, absence of lymphoid tissue in the spleen, signs of tubular epithelial alterations in kidney and any nonspecific change seen in myocardium like mild edema/fiber separation. Images were taken of representative fields using an Olympus EP50 digital system with cellSens software. All findings were recorded and at the end of necropsy compared with gross lesions and qPCR results to determine how each organ was affected by virus in rank order.

### Statistical analysis

All statistics were analyzed with the SPSS (version 25.0, IBM Corp., Armonk, N.Y., USA). Organ-to-body weight ratios, Ct values and derived viral copy numbers were presented as mean ± SD for each dataset. To compare the organ weights between infected and control groups we employed independent-sample t-tests. For the qPCR data (it compared multiple organs), a one-way ANOVA was used. The infected group was compared by Tukey’s post-hoc test when differences within this group had to be analyzed and with the non-infected corresponding controls directly for infection of organs by Dunnett’s test. All tests were 2-sided, and p-value less than 0.05 was considered statistically significant.

## Results

### Infection increases liver and spleen mass and alters tissue-specific viral loads

**Fig 1:**
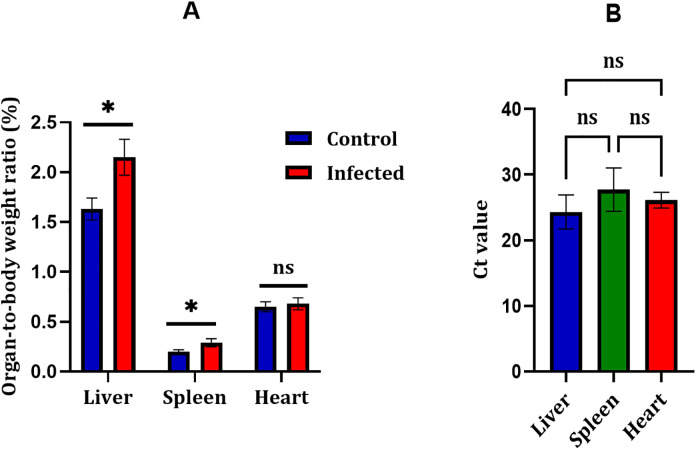
Organ-to-body weight ratios and qPCR Ct values in broiler chickens experimentally infected with FAdV-8a at 21 days post-infection (dpi). (A) Liver, spleen, and heart weights normalized to body weight in infected and control birds. The organ-to-body weight ratios were significantly higher in infected birds for both the liver (*p* = 0.0129) and spleen (*p* = 0.0252), whereas no significant difference was observed for the heart (*p* = 0.5422). Data are presented as mean ± SD (n = 7 per group). (B) Ct values obtained by qPCR targeting the hexon gene in liver, spleen, and heart tissues. The liver exhibited the lowest Ct values, indicating the highest viral load, whereas the spleen and heart showed comparatively higher Ct values. Statistical analysis using one-way ANOVA followed by Tukey’s multiple-comparison test did not reveal significant differences among the examined organs within the infected group (*p* > 0.05). Data are presented as mean ± SD (n = 5).

The liver and spleen from the infected birds showed enlargement when compared with the controls at 21 days post-infection. This was also reflected in the liver-to-body and spleen-to-body weight ratios, which were higher in the infected birds than in the uninfected group, with *p* values of 0.0129 and 0.0252, respectively. In contrast, the heart-to-body ratio did not show a noticeable change, and the difference between groups was not significant (*p* = 0.5422). These findings are illustrated in Fig 1A.

qPCR analysis showed differences in the distribution of viral DNA among the examined organs. In general, lower Ct values were obtained in the liver, indicating higher viral DNA levels, whereas the spleen and heart showed relatively higher Ct values. Although this trend was observed, statistical analysis using one-way ANOVA followed by Tukey’s multiple comparisons test did not reveal significant differences between organs (*p* > 0.05). Even so, the pattern may suggest a tendency toward preferential viral replication in hepatic tissue. Ct values for the examined organs are presented in Fig 1B, and the individual Ct values are provided in Supplementary S2 Table. No mortality or obvious clinical signs were recorded during the experimental period (Supplementary S1 Table).

### Gross pathological alterations following FAdV-8a infection

**Fig 2:**
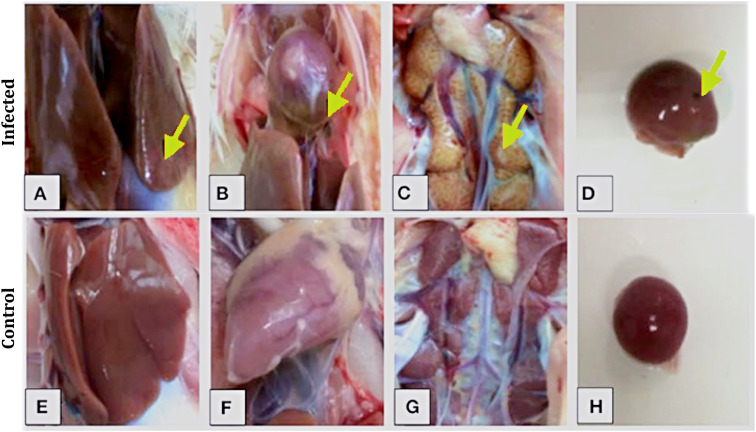
Gross lesions observed in broiler chickens infected with FAdV-8a at 21 dpi. (A) Liver of an infected bird showing enlargement and pale discoloration with scattered mottling. (B) Heart displaying mild pericardial congestion with a slightly opaque epicardium. (C) Kidney with diffuse enlargement, pale cortex, and a coarse reticulated appearance. (D) Spleen showing marked splenomegaly with a dark red congested appearance. Yellow arrows indicate the main areas of pathological change. (E–H) Corresponding organs from control birds showing normal size, shape, and coloration with no visible lesions.

At day 21 post-infection, several gross lesions were observed in the infected birds, whereas these findings were not seen in the control birds. The most common change was an enlarged and pale liver. In many birds, the liver also showed areas of mottling or congestion. This type of appearance is indicative of adenoviral hepatitis and is shown in Fig 2A. Changes in the heart were less marked. In some infected birds, mild congestion of the pericardium was noted, and the myocardium appeared slightly dull compared with normal hearts, although these changes were subtle. These findings are shown in Fig 2B. The kidneys in the infected group showed mild gross changes. They appeared swollen and unusually pale, and the cortical surface often had a coarse or reticulated look. These findings are visible in Fig 2C. The spleen showed the most obvious enlargement. Almost all infected birds had dark-red, congested spleens, and the organ was visibly larger than in control birds, as seen in Fig 2D. In contrast, all the organs collected from the control group looked completely normal. Their liver, heart, kidneys, and spleen showed the expected color and size, with no signs of swelling or discoloration. These normal appearances are shown in Fig 2E–H for comparison.

### Histopathological alterations in FAdV-8a–infected chickens

**Fig 3:**
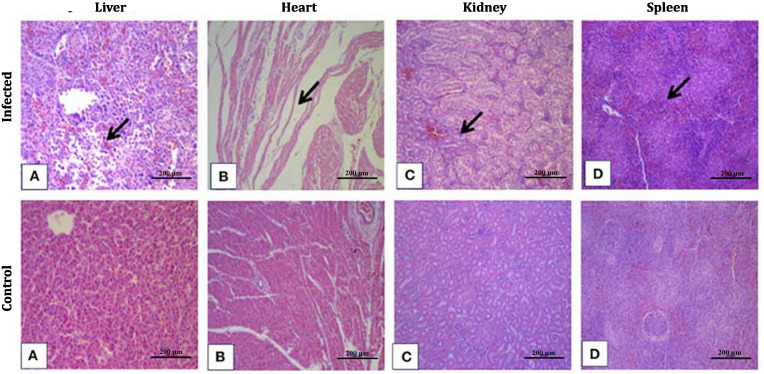
Histopathological lesions in broilers infected with FAdV-8a at 21 dpi. Hematoxylin and eosin–stained sections from infected (A–D) and control (E–H) birds. (A) Liver showing multifocal hepatocellular degeneration and necrosis with mononuclear inflammatory infiltration. (B) Heart showing slight separation of myocardial fibers and interstitial edema. (C) Kidney showing focal congestion and tubular epithelial degeneration. (D) Spleen showing lymphoid depletion and multifocal congestion. (E–H) Representative tissues from control birds with normal histological architecture. The images highlight the characteristic morphological changes observed in organs infected with FAdV-8a. Scale bars = 200 µm.

The microscopical study served to explain this early organ involvement at post-mortem. Multiple areas of hepatocellular degeneration and necrosis were the most uniform lesions found in livers of affected birds. Many of these foci were encircled by a mononuclear inflammatory infiltrate, and the normal tissue architecture was considerably attenuated. These typical hepatic changes are also well visualized in Fig 3A. The heart lesions were far less severe. Foci of myocytes and multifocal interstitial edema with sparse separation of myocardial fibers, but without significant structural changes were observed in most sections. These minor heart changes are depicted in Fig 3B. Renal sections showed that the kidneys were also involved, though to a lesser extent. The most frequent pathological change observed was degeneration of the tubular epithelium, along with small congested areas and mild inflammatory infiltration. This is illustrated in Fig 3C. The spleen showed more noticeable alterations. The white pulp areas showed loss of lymphocytes, and in several sections congested regions were also present. These changes in the spleen are shown in Fig 3D.

Tissues collected from the control group appeared normal. The liver, heart, kidneys, and spleen from control birds maintained their usual microscopic appearance, and no obvious abnormalities were detected. These normal histological structures are shown in Fig 3E–H for comparison.

### Viral DNA detection and quantification in infected organs

**Fig 4:**
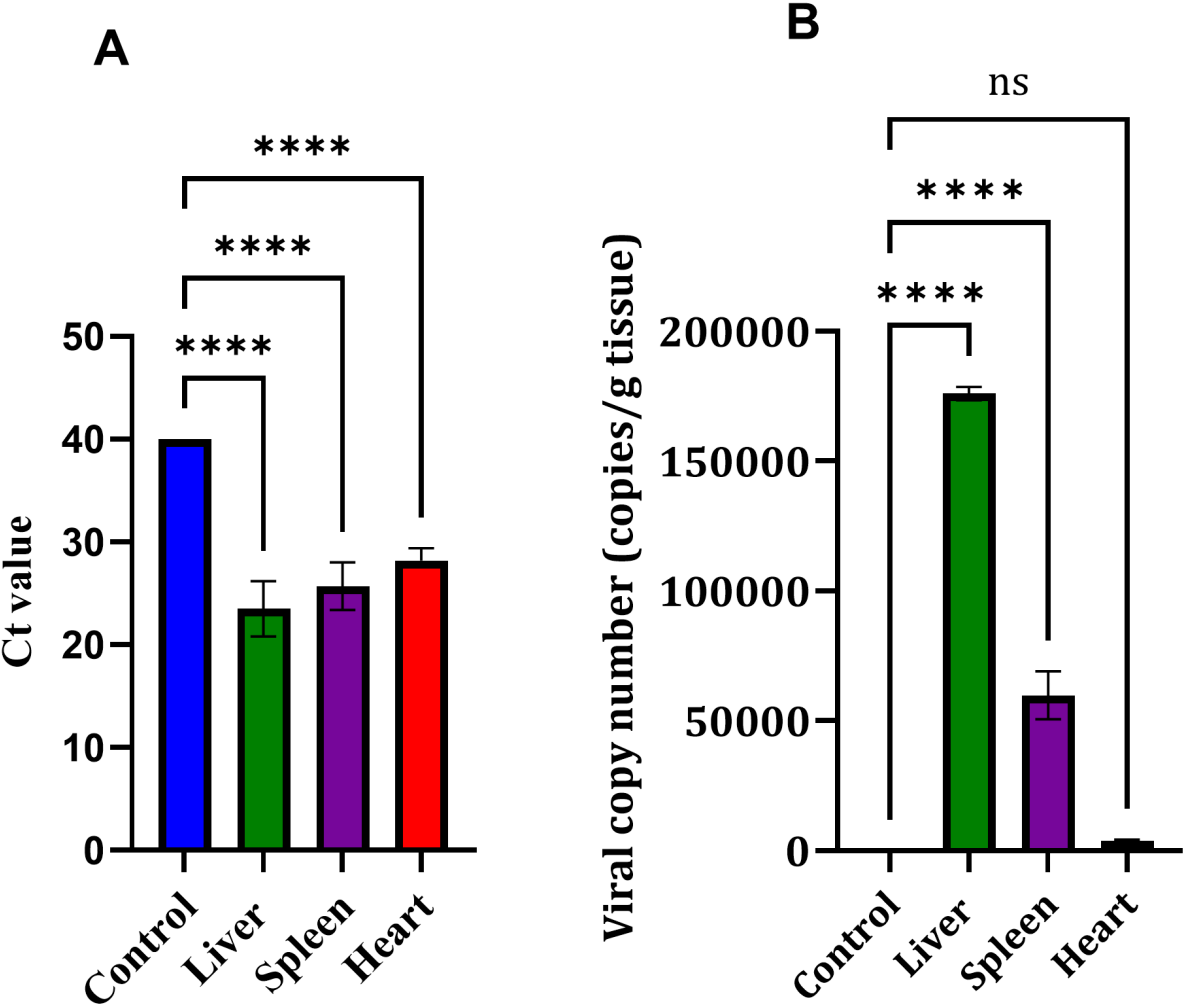
Quantitative detection of FAdV-8a DNA in liver, spleen, and heart tissues at 21 days post-inoculation. (A) Ct values obtained by qPCR analysis. Viral DNA was clearly detected in infected tissues, with significantly lower Ct values in the liver, spleen, and heart compared individually with the control group (*p* < 0.0001 for all comparisons; one-way ANOVA followed by Dunnett’s multiple comparison test). No statistical comparisons were performed among infected organs. Data are presented as mean ± SD (n = 5). (B) Viral genome copy numbers calculated from the qPCR standard curve. The liver contained the highest viral load (~1.7 × 10⁵ copies g ⁻ ¹ tissue), followed by the spleen (~6.03 × 10⁴ copies g ⁻ ¹), whereas only minimal viral DNA was detected in the heart (~2.54 × 10³ copies g ⁻ ¹). Data are shown as mean ± SD (n = 5).

Distribution of viral DNA in different tissues was assessed by quantitative PCR. Differences in Ct values between infected birds (n = 5) and their controls were observed depending on the examined tissue (Supplementary S2 Table). The liver showed the lowest Ct values, indicating the highest viral burden, and this differed significantly from the control group (*p* < 0.0001, Dunnett’s test). Ct values from the spleen and heart were also significantly lower than those of their respective controls (*p* < 0.0001), confirming the presence of viral DNA in these organs. However, Ct values in the spleen and heart were still higher than those observed in the liver. These results are shown in Fig 4A.

Ct values were converted to genome copy numbers using the standard curve generated during the qPCR assay. This allowed estimation of viral DNA levels in each organ. As shown in Fig 4B, the liver contained the highest amount of viral DNA, with an average of approximately 1.76 × 10⁵ copies per gram of tissue. The spleen showed moderate levels (about 5.98 × 10⁴ copies per gram), whereas only low levels were detected in the heart (approximately 3.74 × 10³ copies per gram). Statistical analysis showed that viral DNA loads in the liver and spleen were significantly higher than those in controls (*p* < 0.0001). The heart, however, did not differ significantly from controls (*p* = 0.4885). Taken together, viral replication appeared to occur mainly in hepatic tissue.

## Discussion

The findings of the present experiment suggest that the Iraqi FAdV-8a field isolate produced a disease pattern resembling inclusion body hepatitis. Among the examined organs, the liver appeared to be the most affected organ. The liver was consistently enlarged, and higher levels of viral DNA were detected in this organ. The most evident microscopic lesions were also observed in the liver. Similar observations have been reported for FAdV species D and E, where the liver is usually described as the main site of viral replication [22–24]. These differences in viral load depending on the organ were similar to those seen before, as the highest viral loads occurred in the liver followed by the spleen, while low viral loads were recorded in the heart. These patterns have been noted earlier in FAdV strains 4, 8a, and 8b [25–28]. Thus, the characteristics of this isolate seem similar to those previously described in the scientific literature, although some degree of variation among studies may be expected. Minor changes in the histology of the heart were seen on some occasions, but there was no significant accumulation of virus DNA in this organ. This result suggests that, in general, the heart is not a favorable location for replication by adenoviruses other than HHS viruses [29–31]. The mild cardiac changes seen in the present study may therefore represent secondary findings. They could be related to systemic infection or general physiological stress rather than direct viral replication, although this cannot be completely ruled out.

Previous experimental studies of FAdV infection have generally concentrated on the acute phase, with sampling commonly performed between 5 and 10 days post-infection, when viral loads are expected to reach their peak in target organs [32,33]. In the present study, however, samples were collected at 21 days post-infection to examine viral persistence and tissue distribution beyond the period of maximal replication, and to better reflect conditions closer to market age. Viewed in this context, the relatively lower viral copy numbers detected here likely indicate partial viral clearance during the later stage of infection rather than an absence of tissue tropism. This interpretation is further supported by the highest viral load observed in the present study, approximately 1.76 × 10⁵ copies per gram of liver tissue, which remained below peak values reported during acute infection in earlier studies [32,33]. Together, these findings are consistent with sampling at a post-peak phase and provide additional insight into the persistence of the Iraqi FAdV-8a isolate.

The renal and splenic findings add further context to how the infection behaved systemically. Although kidneys were not included in the organometric or molecular analyses, the tubular degeneration and congestion observed histologically may represent secondary pathological changes, as reported in previous adenoviral infection studies [34,35]. The spleen, in contrast, showed clear lymphoid depletion in many birds. Similar splenic alterations have been reported in previous studies and are often linked to a temporary disturbance of immune tissues during adenoviral infection. The absence of mortality in the present experiment is also consistent with earlier controlled studies in which birds were maintained under optimal conditions without the additional stressors or co-infections that commonly aggravate disease outcomes in commercial production systems [23,36]. Overall, the disease pattern observed in this study resembles that reported from neighbouring countries. Studies from Iran, Egypt and Turkey showed that the majority of regional isolates of FAdV-8a cause severe liver damage; however, high mortality is not always achieved by these isolates [16,37,24]. Such similarity implies that the Iraqi strain we studied may possess some biological traits in common with other circulating Middle Eastern strains. Given that vertical transmission still represents an important way to introduce FAdVs on broiler farms [38], the high rate of hepatic replication observed in this study confirmed that surveillance should be enhanced at the level of the breeder and biosecurity kept stringent. From a control point of view these are well known difficulties. Cross-immunity between adenoviral serotypes is low, and vaccination has to take the circulating serotypes into consideration for meaningful effects. Even though vaccines for serotypes 4, 8b, and 11 have demonstrated promising outcomes others which are serotype-specific for 8a is still not available in several areas of the world such as some regions from Middle East [11,39]. The data generated here may assist in the planning of future vaccine trials and aid in understanding local viral diversity. There are several strengths to this study, including that experiments were performed in a controlled environment, starting point was from a defined time period of sampling, and evidence presented on gross histological and molecular levels. However, there are several limitations that need to be considered. We did not assess for viral load in the kidneys, thus we are unable to make definite conclusions on mechanism of renal injury. In addition, a single field isolate was evaluated. As there is genetic variation in FAdV-8a strains reported from around the world, it may be beneficial to analyze several isolates for future studies to determine the extent of lesions and replication patterns. In conclusion, the disease picture with predominant liver involvement, mild to moderate changes in the spleen and milder effects on the kidney as well as heart were observed during infection of chickens by Iraqi FAdV-8a. It should be noted that sampling was performed at 21 dpi to assess the persistence and tissue distribution of viral DNA during the later phase of infection. Future studies including earlier time points would provide additional insight into the replication kinetics and acute pathological changes associated with FAdV-8a infection. The pronounced hepatic tropism observed in this study is consistent with earlier reports describing the liver as the primary site of replication for FAdV species D and E.

## Conclusion

The findings of the present study indicate that the Iraqi FAdV-8a field isolate produced a disease pattern mainly affecting the liver, with additional involvement of the spleen and mild renal changes, while the heart showed only minor alterations. The liver consistently exhibited the highest viral load together with lesions compatible with the classical pathological features of inclusion body hepatitis. The spleen showed a moderate viral presence accompanied by mild lymphoid depletion, whereas no substantial changes were observed in the kidneys or heart.

No mortality was recorded during the experiment, suggesting that this isolate is of moderate virulence compared with more pathogenic strains, particularly under stress-free conditions and in the absence of concurrent infections. Taken together, these observations provide region-specific information on the behaviour of FAdV-8a in broiler chickens in Iraq. The results also highlight the importance of continued monitoring, especially in relation to vertical transmission and the limited cross-protection reported among adenovirus serotypes. The disease pattern observed here further supports the value of targeted surveillance at the breeder level and points to the potential benefit of serotype-specific preventive strategies, including vaccines based on locally circulating strains.

## Supporting information

S1 Table Daily monitoring records for the remaining birds during the experimental period. (XLSX)

S2 Table Raw Ct values for individual birds used in qPCR analysis. (XLSX)
